# Cross-species hybridizations on a multi-species cDNA microarray to identify evolutionarily conserved genes expressed in oocytes

**DOI:** 10.1186/1471-2164-7-113

**Published:** 2006-05-10

**Authors:** Maud Vallée, Claude Robert, Steve Méthot, Marie-France Palin, Marc-André Sirard

**Affiliations:** 1 Centre de Recherche en Biologie de la Reproduction, Département des Sciences Animales, Université Laval, Québec, G1K 7P4, Canada; 2 Dairy and Swine Research and Development Center, Agriculture and Agri-Food Canada, Lennoxville, Québec, J1M 1Z3, Canada

## Abstract

**Background:**

Comparative genomic analysis using cDNA microarray is a new approach and a useful tool to identify important genetic sequences or genes that are conserved throughout evolution. Identification of these conserved sequences will help elucidate important molecular mechanisms or pathways common to many species. For example, the stockpiled transcripts in the oocyte necessary for successful fertilization and early embryonic development still remain relatively unknown. The objective of this study was to identify genes expressed in oocytes and conserved in three evolutionarily distant species.

**Results:**

In this study we report the construction of a multi-species cDNA microarray containing 3,456 transcripts from three distinct oocyte-libraries from bovine, mouse and Xenopus laevis. Following the cross-species hybridizations, data analysis revealed that 1,541 positive hybridization signals were generated by oocytes of all three species, and 268 of these are preferentially expressed in the oocyte. Data reproducibility analyses comparing same-species to cross-species hybridization indicates that cross-species hybridizations are highly reproducible, thus increasing the confidence level in their specificity. A validation by RT-PCR using gene- and species-specific primers confirmed that cross-species hybridization allows the production of specific and reliable data. Finally, a second validation step through gene-specific microarray hybridizations further supported the validity of our cross-species microarray results. Results from these cross-species hybridizations on our multi-species cDNA microarray revealed that *SMFN *(Small fragment nuclease), *Spin *(Spindlin), and *PRMT1 *(Protein arginine methyltransferase 1) are transcripts present in oocytes and conserved in three evolutionarily distant species.

**Conclusion:**

Cross-species hybridization using a multi-species cDNA microarray is a powerful tool for the discovery of genes involved in evolutionarily conserved molecular mechanisms. The present study identified conserved genes in the oocytes of three distant species that will help understand the unique role of maternal transcripts in early embryonic development.

## Background

**Figure 1:**
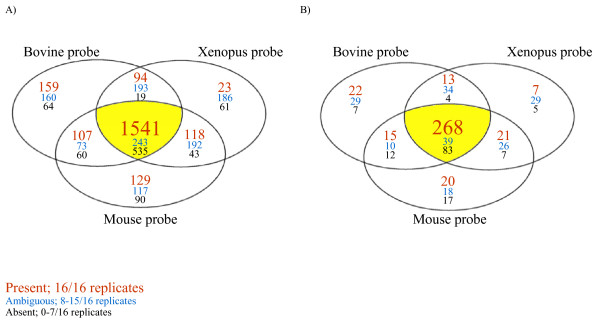
**Venn diagram representing clones present in oocytes of one, two or all three species**. Clones are considered as present when their signal intensities are above threshold in every replicates (16/16), they are considered as ambiguous when their signal intensities are above threshold in 8 to 15 replicates, and finally are considered absent when their signal intensities are above threshold in less than 7 replicates. A) All the clones on the microarray slides are considered, B) only the clones preferentially expressed in the oocyte are considered. For the clones present in all three species, the number of clones derived from each oocyte library is given in table 1.

Evolutionarily distant animals exhibit common mechanisms and pathways involved in early development. One of the characteristics conserved across species is that the oocyte arrests during the first meiotic division, where a stockpile of transcripts and proteins that are synthesized and stored will subsequently support early development [1,2]. The maternal transcripts that are stored in the oocyte will drive meiotic resumption of the oocyte and early cleavage divisions of the embryo up to zygotic genome activation [3]. In Xenopus, major zygotic genomic activation takes place after 12 rapid synchronous cleavage divisions generating > 4,000 cells, while in the bovine and mouse it occurs at the eight- to sixteen-cell stages and two-cell stage, respectively [4-6]. It is speculated that several hundred maternal transcripts play an active role in early development, although only a few have been identified to date [7]. Information for only a limited number of these genes is currently known, meaning that our basic understanding of gene expression patterns driving pre-implantation development is still very restricted. A few maternally expressed genes with important functions related either to oogenesis, folliculogenesis, fertilization, and or early embryonic development have been discovered in the mouse oocyte, such as *Mos *(Moloney sarcoma oncogene) [8], *Zp3 *(Zona pellucida glycoprotein 3) [9], *Zp2 *(Zona pellucida glycoprotein 2) [10], *Zp1 *(Zona pellucida glycoprotein 1) [11], *Gdf9 *(Growth differentiation factor 9) [12], Fig 1*α *(Factor in the germline alpha) [13], *Bmp15 *(Bone morphogenetic protein 15) [14], *H1foo *(H1 histone family, member O, oocyte-specific) [15], *Zar1 *(Zygote arrest 1) [16], *Mater *(Maternal antigen that embryos require) [17], *Npm2 *(Nucleophosmin/nucleoplasmin 2) [18], and *Msy2 *(Y box protein 2) [19]. Some of these oocyte-specific genes have been identified via model organisms; for instance the mammalian oocyte-specific cleavage stage linker histone *H1foo *and the *Msy2 *gene were both first identified in the Xenopus laevis oocyte [15,19]. Therefore, our ability to compare the conserved maternal genes across evolutionarily distant species that share common mechanisms, such as the Xenopus laevis, mouse and the bovine, will contribute by identifying functionally important genes involved in early development.

In the past, embryonic development has been studied through time-consuming gene-by-gene analyses that characterized only very specific molecular mechanisms. The need for large-scale genomic approaches is required to analyze a large cohort of genes simultaneously. Suppressive subtractive hybridization (SSH) and differential display (DDRT) have been successfully applied to early developmental studies [20-25]. Analysis of expressed sequence tags (EST) has also been used to study the gene expression that occurs during early development [7,26]. Furthermore, the large amount of sequence information that has been placed into public databases over the last decade has allowed for the use of In Silico approaches to identify oocyte-specific transcripts [27,28]. In Silico approaches are ideal for mouse studies due to the large quantity of genomic information available for this species. Unfortunately, genomic information is quite limited for bovine and Xenopus, which renders this approach less feasible in this case. Thus, when the objective is to compare those three species, the In Silico analysis cannot be used as the main approach, but the information that it can provide can help support as in this case, the microarray results. Recently, microarrays have been widely used for large-scale transcriptome analyses and have proven to be a powerful approach to study molecular mechanisms underlying early development [24,29-33]. However, DNA arrays are currently available for only a limited number of species and to overcome this limitation, cross-species hybridization has been utilized as one potential solution. For example, human arrays have been used to study gene expression patterns in both the swine and bovine species [34,35]. Gene expression profiles have been compared between the human and canine, pig, bovine, and chimpanzee using human arrays [29,36-39]. While studies using cross-species hybridization have used different platforms (oligo or cDNA arrays), they have all used arrays that were designed based on the sequence of only one species. In an attempt to address this cross-species issue, another study constructed a multiprimate cDNA array to study the effect of sequence divergence on gene expression analysis [40]. It is now possible to apply microarrays beyond the conventional usage, proving that this technique can be flexible, however one has to be careful and respect the limits of cross-species hybridization.

Here we report the construction and hybridization of a multi-species cDNA array representing 3,456 oocyte transcripts from bovine, mouse and Xenopus laevis. A total of 1,152 clones from each species were randomly selected from three distinct oocyte-subtracted libraries generated through SSH for the construction of this multi-species cDNA microarray. Hybridizations allowed the identification of candidate genes conserved in those three species and those candidates that are preferentially expressed in the oocyte. We show herein that cross-species comparison using these arrays is a powerful tool for the discovery of evolutionarily conserved molecular mechanisms related to the unique genes and functions found in the oocyte.

## Results

### Microarray data analysis

#### Transcripts present in oocytes

The transcripts considered as present in the bovine, mouse or Xenopus laevis oocytes, were selected based on the following criteria. Normalized and log transformed data points above a calculated threshold were considered as "present". The threshold was calculated with the intensity values of the 424 negative control spots present on the array. 75% of the clones showed signal intensity above the threshold, thus considered as expressed in oocytes of at least one species. The distribution of the expressed clones was as follows; 35% bovine clones, 31% mouse clones and 34% Xenopus laevis clones. The percentage of expressed clones is in close agreement with the percentage of insert-containing clones found in the libraries spotted on our array [24].

#### Transcripts common in oocytes of all three species

The clone distribution for all the clones on the array is presented in a Venn diagram according to their detection in oocytes of one, two, or three species based on a three level score (present, ambiguous, absent) (Fig. 1A). Transcripts that were above the calculated threshold for all data points (48 data points were generated for each clone; 4 spotted clones × 4 hybridizations replicates × 3 species) were selected as transcripts present in oocytes of all three species (Table 1, Fig. 1A). These analyses revealed that 45% of the transcripts (1,541) gave hybridization signals above the calculated threshold, thus were considered as present in oocytes from all three species. More specifically 718 transcripts are from the bovine oocyte library, 476 and 347 transcripts are from the mouse and Xenopus oocyte libraries, respectively (Table 1).

#### Oocyte-specific transcripts common in all three species

To further characterize this subpopulation of genes conserved in all three species, another classification was performed in order to identify the ones that are preferentially expressed in oocytes. This was done by comparing results from a previous study [24] where transcripts preferentially expressed in oocytes compared to somatic tissues were identified. In this previous study, a list of genes preferentially expressed in the oocyte was obtained through subtractive hybridization and microarray experiments in the bovine, mouse and Xenopus laevis. For the present study, the genes found to be conserved in all three species (1,541) were compared against the list of genes preferentially expressed in the oocyte previously obtained. The comparison of these two lists revealed that 268 clones are preferentially expressed in oocytes and also conserved across all three species (Table 1, Fig. 1B). That list can be further subdivided as follows; 120 transcripts originate from the bovine oocyte library, 96 from the mouse oocyte library, and 52 from the Xenopus oocyte library (Table 1). A representative list of transcripts preferentially expressed in the oocyte and conserved across species is presented in table 2.

#### TMeV visualization of microarray data

**Figure 2:**
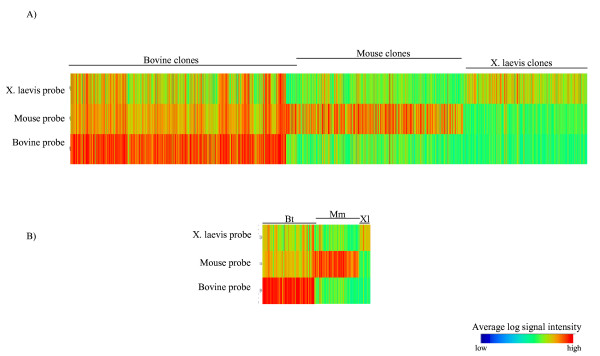
**TIGR Multiexperiment Viewer representation of microarray data**. Transcripts were ordered horizontally by clone number and grouped by species. Average log signal intensities are presented for clones hybridized with probes corresponding to one of the three species. The expressed clones that are conserved across all three species are represented in (A) and those conserved across species and preferentially expressed in the oocyte are represented in (B).

To better visualize the microarray results, a versatile microarray data analysis tool, TIGR Multiexperiment Viewer (TMeV), was used. The average normalized log intensities for the 1,541 transcripts listed above are represented in figure 2A. Results are shown for each probe corresponding to one of the three species, and transcripts were ordered manually by clone number. Clearly evident with this representation is the fact that same-species hybridizations produced globally higher signal intensities than cross-species hybridizations. However, the cross-species hybridizations also generated signal intensities that were visibly and significantly above background. Also, bovine clones generate higher signal intensities than clones from the other two species, independent of the probe used for the hybridization. This is mainly a methodological artifact as the spotted bovine clones were more concentrated than the others (average concentration of spotted clones; bovine: 157 ng/ul, Mouse: 55 ng/ul, and Xenopus laevis: 59 ng/ul). Also, it was not due to a probe effect since this was controlled with our positive control, a cDNA fragment of the Green Fluorescent Protein (*GFP*). Figure 2B shows the 268 transcripts considered as genes preferentially expressed in oocytes and conserved in all three species.

### Analysis of cross-species versus same-species hybridization

#### Reproducibility

An essential criterion for the application of cross-species experiments is data reproducibility. To test this, we calculated the correlation coefficients of signal intensities between replicated experiments in a pair-wise manner (Table 3). For each experiment, the signal intensities generated from a probe corresponding to one species was calculated by comparing the signal intensities obtained from a replicate experiment. The correlation coefficients were first calculated with the signal intensities of all clones on the microarray slides. Next, the correlation coefficients were calculated with the signal intensities of clones corresponding to one species at a time, thus generating CC for same-species and cross-species hybridizations (Table 3). Results show that when all the spots on the multi-species microarray are considered, the CCs are relatively high and very similar between the three species (0.934 – 0.957). The analysis also revealed that Xenopus laevis clones generate the lowest CCs even with the Xenopus laevis probe (0.852). Taking this into consideration, the CCs calculated with data from cross-species hybridizations are in an acceptable range (0.817 – 0.965).

#### Same species versus cross-species hybridizations

**Figure 3:**
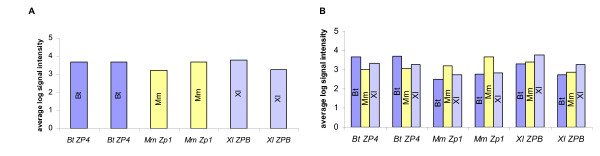
**Same-species versus cross-species hybridization**. The hybridization pattern of one member of the *Zp *gene family is represented. Average log signal intensities for two different clones corresponding to the same *Zp *genes from each species for A) same-species hybridization and B) cross-species hybridization. Probe species are indicated inside each bar. Bt; Bos taurus, Mm; Mus musculus, Xl; Xenopus laevis.

**Figure 4:**
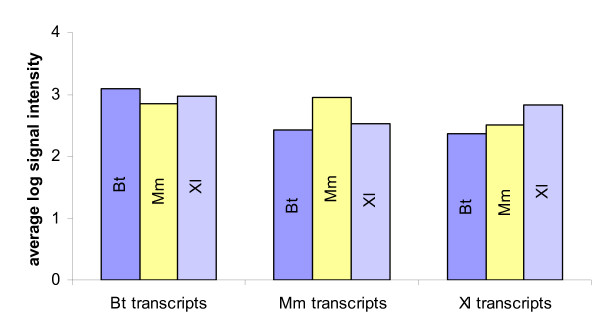
**Average log signal intensity for all 3,456 clones**. Shown here are average global log signal intensities values for same-species and cross-species hybridization. Results are separated according to the respective species of the clone in order to compare same-species versus cross-species hybridization. Probe species are indicated inside each bar. Bt; Bos taurus, Mm; Mus musculus, Xl; Xenopus laevis.

After assessing the level of data reproducibility, variations between same-species and cross-species hybridizations were analyzed. A candidate gene, member of the *Zp *family (Bt; *ZP4*, Mm; *Zp1*, Xl; *ZPB*), was selected according to its presence in all three libraries, its previously reported expression in oocytes of the three species being studied, and its evolutionary conserved sequence [Homologene: 33483]. By comparing between hybridizations the average log signal intensities of two clones from each species corresponding to the same *Zp *family member (n = 6), it is possible to observe that all the clones corresponding to this gene are consistent, not only between same-species, but also among the three different species (Fig. 3A). In cross-species hybridizations, all cross-species hybridizations signals are weaker than same-species hybridization signals, but are still relatively high, and always significantly above background (Fig. 3B). A similar analysis was performed with all 3,456 transcripts found on our multi-species microarray (Fig. 4). Once again, as anticipated, same-species hybridizations result in higher signal intensities than cross-species. Nevertheless, cross-species hybridizations average signal intensities are above background intensity suggesting that cross-species hybridizations are specific.

#### In Silico sequence similarity analysis

Another essential criterion for successful cross-species hybridization is adequate homology. To assess the extent of this potential problem, all transcripts identified as being preferentially expressed in the oocyte in all three species were compared to identify possible hits against the other two species using the GenBank database (Table 4). On average, bovine sequences show an 86% and 80% identity rate with the mouse and Xenopus laevis sequences, respectively and between the mouse and Xenopus, an 80% identity rate is observed. The BLAST results in average were given for a target region of an acceptable length (average 243 nt). However, not all transcripts compared against the GenBank database gave positive hits (Table 5). For the mouse, 64% of the transcripts gave no significant positive hit when compared to the other two species, whereas 41% of the Xenopus laevis and 29% and bovine transcripts also resulted in no significant positive hit. Amongst the Xenopus transcripts analyzed, 48% gave positive hits for the other two species. As for bovine and mouse it was even lower, where 37% and 12% of the transcripts gave positive hits for the other two species.

### Validation of cross-species hybridization specificity

In order to support the results obtained with our microarray analysis, we performed a two-step validation process. First, this validation consisted of a standard detection test using RT-PCR and secondly a microarray hybridization analysis using specific PCR products as probes. Three candidates were selected for this validation process, one for each species, based on criteria originating from the microarray results. First the transcript had to generate a positive hybridization signal in all three species (in all 16 repetitions) and had to be preferentially expressed in the oocyte. Next, the candidate had to have a known ortholog gene in the two other species, an essential criterion if we wanted to design species-specific primers. Finally, we selected candidates that were not yet reported to be expressed in the oocyte for the two other species since we wanted to know if this cross-species hybridization approach allowed the production of precise and reliable data across species that could lead to the discovery of novel transcripts present in the oocyte.

#### RT-PCR amplification

**Figure 5:**
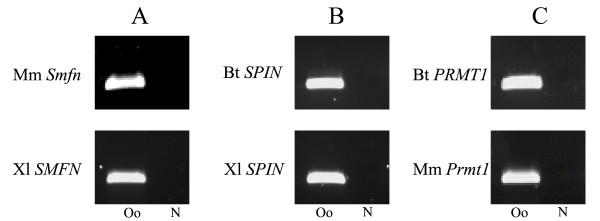
**Validation of microarray result by simple detection analysis using RT-PCR for three candidate transcripts**. Amplifications are performed in oocytes of two species with gene- and species-specific primers designed according to the known sequences in respective species. A) Amplification in mouse and Xenopus laevis oocytes of *SMFN *transcript originally found in the bovine oocyte-library. B) Amplification in bovine and Xenopus laevis oocytes of *Spin *transcript originally found in the mouse oocyte-library. C) Amplification in bovine and mouse oocytes of *Prmt1 *transcript originally found in the Xenopus laevis oocyte-library. Bt; Bos Taurus, Mm; Mus musculus, Xl; Xenopus laevis, *Smfn*; Small fragment nuclease, *Spin*; Spindlin, *Prmt1*; Protein arginine methyltransferase 1, Oo; oocyte, N; negative control.

The RT-PCR amplifications were performed on cDNA generated from oocytes total RNA using gene- and species-specific primers (Table 6). For each candidate gene, the amplifications were performed on the other two species, not the species in which the candidate was first identified since the expression in the oocyte was already known for this species. The three candidate genes selected for validation process were SMFN (Small fragment nuclease) from the bovine oocyte-subtracted library, Spin (Spindlin) from the mouse oocyte-subtracted library and PRMT1 (Protein arginine methyltransferase 1) from the Xenopus laevis oocyte-subtracted library. This RT-PCR validation process revealed that all three candidates showed amplification products in oocytes of the other two species (Fig. 5). Resulting amplicons were sequenced to check for specificity. Identity was compared between the sequences of the PCR amplicons and the clone found on the array, and an acceptable identity rate was obtained (average 85%, min 76 %, and max 94%). Thus further supporting our multi-species microarray results.

#### Gene-specific microarray hybridization

**Figure 6:**
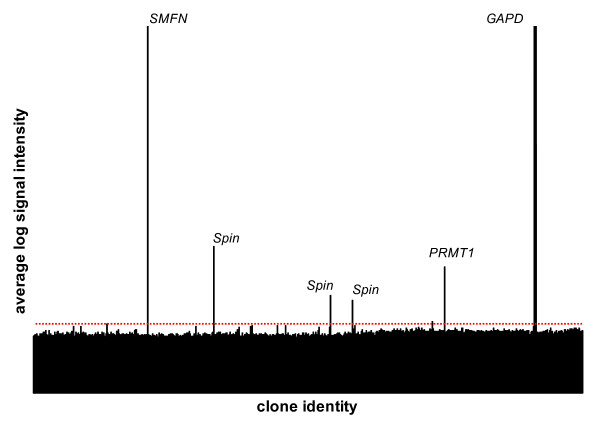
**Gene-specific cross-species hybridization on our multi-species array**. Example of a representative result for average log signal intensities is shown for all the 3,456 transcripts on the array. The red dotted line represents background intensity. The three candidate genes and our positive control *GAPD *are identified. Note that *Spin *transcript is present 3 times on the multi-species microarray. *Smfn*; Small fragment nuclease, *Spin*; Spindlin, *Prmt1*; Protein arginine methyltransferase 1.

The second step in this validation process was to perform gene-specific microarray hybridization on the multi-species cDNA microarray. Following the RT-PCR amplification reaction, the amplicons were labeled and hybridized to our multi-species microarray slide in order to assess the efficiency of gene-specific but cross-species hybridizations. This experiment was performed in three replicates and hybridizations were performed with either probes of both species simultaneously or only one species at a time. We reasoned that the validation could be considered successful only if the signals detected significantly above background corresponded to the selected candidate spotted on the array. This validation process revealed that all three candidates showed specific cross-species hybridization on our multi-species microarray slide supporting once again the idea that our cross-species hybridizations are specific even between not so closely related species (Fig. 6).

## Discussion

The microarray gene chip platform is a powerful tool allowing for the analysis of thousands of genes simultaneously. In this study, we explored the technical feasibility of utilizing cross-species hybridizations to identify genes expressed in the oocyte and that are conserved across three species. Our experimental strategy was twofold. The first goal was to test the possibility of cross-species hybridization of three distantly related vertebrates on cDNA arrays, and most importantly, our second goal was to identify genes expressed in oocytes of all three species. The rationale was to use a subtractive strategy to produce libraries enriched in transcripts preferentially expressed in oocytes, and to use the homology requirement for high specificity microarray hybridization to identify those transcripts that are conserved through evolution. Moreover, we believed that transcripts fulfilling both criteria, specificity to the oocyte and evolutionarily conserved, are potentially important maternal genes involved in key functions of oocyte maturation and early development. By working simultaneously with three evolutionarily distant species, it increased our efficiency at identifying novel oocyte-specific genes and elucidating the important evolutionarily conserved mechanisms in different species. Also, this approach facilitates the identification of new genes not previously identified due to their low expression level in a particular species.

One of the unique features of preimplantation embryo development is that it occurs in the presence of maternally stored RNAs in oocytes as the embryonic genome has yet to be activated. These transcripts have specific functions either in oogenesis, oocyte maturation, fertilization and/or the early phase of preimplantation development. Only a few of these genes are well known and have been characterized, as Sharov et al., have demonstrated that, in the mouse, 119 out of the 196 oocyte-specific ESTs were unknown genes in 2003 [7]. Identification and characterization of these genes will enable us to better understand the unique molecular mechanisms present in the oocyte.

In the present study we designed a cDNA multi-species microarray containing 3,456 transcripts from bovine, mouse, and Xenopus laevis oocytes. Transcripts found on the array were randomly selected from oocyte-subtracted libraries constructed in a previous study [24]. The use of clones coming from three different subtracted libraries created a variation between the three species in the average concentration of the clones spotted on the slides. Based on our multi-species cDNA microarray results, 1,541 transcripts in total gave positive hybridization signals in oocytes across all three species and of these, 268 transcripts in total were found to be preferentially expressed in oocytes for all three species. However, the higher concentration of the spotted bovine clones resulted in more bovine clones being identified as conserved in oocytes of all three species when compared to the number of identified mouse and Xenopus laevis clones. The difference in the concentration of the spotted clones also explains the general higher signal intensities seen in bovine clones. However, although this created a distortion in species representation in the cross-species hybridization results, the average concentration of spotted clones is within acceptable range for each species according to our quality control experiment and the slides manufacturer. The distribution of clones considered as present is practically equal between the three species (35%, 31% and 34% for bovine, mouse, and Xenopus laevis clones respectively). This methodological artifact might have resulted in a failure of identifying all transcripts conserved across species but nevertheless did not affect the validity of the results obtained.

In this study, results obtained from the reproducibility analysis increased the confidence in the data generated from our hybridizations. The CCs calculated with all the clones on the array showed that signal intensities from all three probes used were highly reproducible (0.934 – 0.957). For the bovine and mouse probes, cross-species hybridizations showed a slightly lower correlation coefficient compared with the same-species experiments. On the other hand, Xenopus laevis clones always generated the lowest CCs even with the Xenopus laevis probe, which might be in part related to a lower amount of cDNA spotted onto the array. Nevertheless, the correlation coefficients for the cross-species hybridization were sufficiently high to assure that reproducibility between replicated experiments is acceptable, increasing our confidence in the validity of cross-species hybridizations results. Also, the sequence mismatches present between the three species should be taken into account since they probably contribute to the lower correlations observed in cross-species hybridizations.

The degree of homology between probes and targets when performing cross-species hybridizations is extremely variable. In the presence of sequence mismatches, relative hybridization intensities will reflect both differences in transcript abundance levels, as well as differences in hybridization kinetics. In addition, it can even be variable between two different cross-species hybridizations, especially when the studied species are not equally divergent. Due to these limitations, the goal of this study was not to assess gene expression levels but instead, to survey the products of three subtracted libraries in order to identify transcripts present in oocytes of all three species. Nevertheless, sequence homology had to be sufficiently high in the target region to result in a proper hybridization, since mismatches will inevitably occur in evolutionarily distant animals. To assess this issue, all transcripts identified as being preferentially expressed in the oocyte and present in all three species were compared to identify possible hits with the other two species using the GenBank database. On average, our bovine sequences showed an 86% and 80% identity rate with the mouse and Xenopus laevis sequences, respectively and between the mouse and Xenopus, an 80% identity rate was observed. In the last few years, a number of studies have successfully used cross-species hybridizations [29,34-40]. Ji et al., created a simple mathematic model for cross-species hybridization and concluded that a contiguous matched oligo of 16 bp long was sufficient to generate a specific hybridization signal [41]. Kane et al., have also reached similar conclusions where their results showed that specificity of the probe requires target-genes to be at least 75% similar over the target region [42]. In addition, if the target region is marginally similar (50–75%), a stretch of complementary sequence of more than 15 contiguous bases will allow hybridization [42]. These studies further support the results obtained from cross-species hybridization. However, it has to be considered that this can also be regarded has a limitation inherent to cDNA arrays, since it may also allow some cross-hybridization with other isoforms and/or non-target transcripts and therefore allow non-specific hybridization signals to contribute to the overall signal. Validation by PCR with gene-specific primers can verify this limitation.

Furthermore, the present study has demonstrated that cross-hybridization results can be confirmed by both RT-PCR reactions and gene-specific hybridizations. In order to ascertain that the observed signals were not originating from the annealing of random non-specific sequences, specificity validation was conducted through a simple detection test using species- and gene-specific primers and by gene-specific microarray hybridization. The labeled amplification products corresponding to three independent single genes showed positive signals only for their corresponding target even across species, lending support to the validity of our cross-species hybridizations.

Three candidate genes, one from each species, were selected for these validation processes. Our first candidate gene used for validation was bovine *SMFN*, also known as *CGI-114*, which is homologue of *Orn*, a 3-prime-to-5-prime exoribonuclease of *E. coli*. The ORN protein is known to attack the free 3-prime hydroxyl group on single-stranded RNA, releasing 5-prime mononucleotides in a sequential manner [GeneID: 25996] [43]. In human, a study by Nguyen et al., suggests a role for *SMFN *in cellular nucleotide recycling [44]. In the mouse, *Smfn *gene has also been characterized and is reported to be expressed in a variety of tissues including testis, uterus and embryo, but to date, no report indicates its expression in mouse oocytes [Unigene: Mm.21911]. The similarity between the mouse *Smfn *gene sequence and our bovine clone sequence is relatively high; 91% on 304 bp. A Blast search of our bovine clone sequence against Xenopus laevis sequence revealed that a cDNA clone IMAGE 7205916 [GenBank: BC087528] has an acceptable identity rate with our bovine clone; 79% on 206 bp. This clone is reported to be testis specific [Unigene: Xl.9259]. Like in the mouse, no expression was reported in the Xenopus laevis oocyte for this transcript. With our cross-species microarray hybridization we were able to detect the presence of *SMFN *transcripts in bovine, mouse and Xenopus laevis oocytes, and this was also confirmed by RT-PCR and gene-specific cross-species microarray hybridizations.

Our second candidate gene used for validation was mouse *Spin *(Spindlin), an abundant maternal transcript present in the unfertilized egg and 2-cell, but not 8-cell mouse embryo [45]. SPIN protein associates with the meiotic spindle and is modified by phosphorylation in a cell-cycle-dependent fashion, and is suggested to play a role in cell-cycle regulation during the transition from gamete to embryo [45]. Also, further studies imply that SPIN is a substrate in the MOS/MAP kinase pathway and that this phosphorylation of Spin may be essential for its interaction with the spindle [46]. The *SPIN *gene has not been identified and characterized thus far in the bovine, however there is a predicted sequence from an automated computational analysis in the NCBI database [GenBank: XM_614403]. This predicted sequence has 94% similarity (234 bp) with our mouse *Spin *clone. Since it is a predicted sequence, no report of its expression pattern is available. A Blast search of our mouse clone sequence against Xenopus laevis sequences revealed that a cDNA clone IMAGE 6324148 [GenBank: BC097748] has an acceptable identity rate with our clone; 84% on 146 bp. No report of the expression pattern for the bovine and Xenopus laevis are currently available. Once again, with our cross-species microarray hybridization we were able to detect the presence of *SPIN *transcripts in bovine, mouse and Xenopus laevis oocytes, and this was also confirmed by RT-PCR and gene-specific cross-species microarray hybridizations.

The third and last candidate gene used for validation is the Xenopus laevis Protein arginine methyltransferase 1 (*PRMT1*), an xCirp2-binding protein. The methylation of xCIRP2 (cold-inducible RNA binding protein 2) by PRMT1 results in the accumulation of xCIRP2 in the cytoplasm [47]. It is also known that xCIRP2, which is highly expressed in Xenopus laevis oocytes, is associated with ribosomes, suggesting that it participates in translational regulation in oocytes [48]. Bovine *HRMTl2 *(Hmt1 hnRNP methyltransferase-like 2) gene possesses a high similarity with Xenopus *PRMT1*; 78% on 546 bp. This bovine gene is reported to be expressed in different tissues such as fetus and adult brain but there is no report available indicating its expression in the oocyte [Unigene: Bt.4871]. Mouse Heterogeneous nuclear ribonucleoproteins methyltransferase-like 2 (*Hrmt1l2*) also possess a high identity rate with Xenopus *PRMT1*; 78% on 413 bp. Mouse *Hrmt1l2 *gene is reported to be expressed in a variety of tissues including testis, ovary and embryo, but there are no reports indicating its specific expression in the oocyte [Unigene: Mm.21911]. Once more, we were able to detect the presence of *PRMT1 *transcripts in bovine, mouse and Xenopus laevis oocytes, and this was also confirmed by RT-PCR and gene-specific cross-species microarray hybridizations.

## Conclusion

In summary, these results prove the feasibility of cross-species hybridization and the utility of a multi-species microarray. Our results demonstrate that cross-species hybridization is not only useful for studying species for which microarrays are not yet available, but are also very powerful in elucidating the important evolutionarily conserved mechanisms in different species. The identification of all genes expressed in oocytes will allow a better understanding of the mechanisms and pathways regulating gametogenesis and embryogenesis.

## Methods

### cDNA Multi-species microarray preparation

The multi-species cDNA microarray used in this study contained transcripts from three oocyte-specific libraries constructed previously using Suppressive Subtractive Hybridization (SSH) [24]. Briefly, total RNA from a pool of somatic tissues was subtracted to total RNA from oocytes to generate three libraries enriched in oocyte-specific transcripts (mouse, bovine, and Xenopus laevis). The complete procedure used for slide preparation has been described previously [24]. The array was strictly divided into three equal sections, each corresponding to one of the three species. There were a total of 3,456 oocyte transcripts represented on the array, thus 1,152 clones per species. Each transcript was spotted four times for a total of 13,824 spots. It is important to note that the libraries did not only consist of unique transcripts, it was possible that more than one sequence and/or several copies of a sequence were present on the slide. In addition, negative and positive controls were randomly distributed on the cDNA multi-species array for diverse quality controls; three different SpotReport Alien cDNA Array Validation System (Stratagene, La Jolla, CA) were used as negative controls (n = 424) and a cDNA fragment of the Green Fluorescent Protein (*GFP*) was used as an exogenous positive control (n = 260).

### DNA sequencing and analysis

DNA sequencing was performed as previously described [24]. The resulting sequence traces were visualized with the online freeware Chromas 1.45 [49] and uploaded into a cDNA Library Manager program (Genome Canada Bioinformatics) that automates and facilitates sequence analysis and clone identification. Briefly, sequence traces were uploaded into the cDNA Library Manager, trimmed (Phred software) and compared against a locally installed GenBank database [50].

### Labeling probes for the cDNA Multi-species microarray

Forward-subtracted PCR products from the subtracted libraries (oocyte minus somatic tissues) corresponding to oocyte-libraries were used as probes to hybridize the cDNA multi-species microarray as previously described [24]. Briefly, probes were labeled with Alexa Fluor 555 and 647 reactive dye packs (Molecular Probe, Burlington, ON, Canada) using Amino Allyle dUTP (Ambion, Austin, TX) according to the manufacturer's instructions.

### Array hybridization

Slides were prehybridized with DIG buffer (Roche Diagnostics, Laval, QC, Canada) supplemented with yeast tRNA (4 mg/ml, Invitrogen) and Cot-1 DNA (1 mg/ml, Invitrogen) for 1 hour at 37°C. Slides were then hybridized overnight at 37°C with labeled purified probes added to fresh prehybridization solution. Hybridizations were performed in the ArrayBooster using the Advacard AC3C (The Gel Company, San Francisco, CA). Slides were then washed once with 1 × SSC-0.2% SDS for 10 min at RT, 1 × SCC-0.2% SDS for 10 min at 55°C, and for 5 min at RT with 0.1 × SCC-0.2% SDS. Hybridizations were always performed with probes corresponding to two different species, in an all pair design (bovine-mouse; mouse-Xenopus laevis; Xenopus laevis-bovine). A dye swap experiments was included for each sample to take into consideration the variation in dye incorporation efficiency. Moreover, two biological replicates for each sample were used. Thus, for each species, the hybridizations were carried out twice with one dye and twice with the other dye, giving four technical replicate hybridizations per species. Since each clone was replicated four times on the microarray, a total of 16 data points were generated for all the candidates per species-specific hybridization.

Considering that the same spots were also hybridized during cross-species hybridizations, 48 data points were generated for each spotted clone (4 spotted clones × 4 hybridizations × 3 species). Quality control was performed through the addition of 424 negative and 260 positive controls included on the array. *GFP *cDNA fragments were added to the probes in equal amounts, before labeling, to use as positive controls.

### Microarray image processing

Slides were scanned using the VersArray ChipReader System (Bio-Rad, Mississauga, ON, Canada) and visualized with the ChipReader software (Media Cybernetics, San Diego, CA). Microarray image processing was performed with the ArrayPro Analyzer software (Media Cybernetics, San Diego, CA). Local background was subtracted and data were normalized (LOWESS). Microarray experiments presented in this study adhere to the standards proposed by the Microarray Gene Expression Data Society [51]. Raw and normalized data for the microarray experiments reported herein are stored in the public repositories ArrayExpress (accession no E-MEXP-488) [52].

### Microarray data analysis

#### Transcripts present in oocytes

First, data were log transformed before proceeding with a simple analysis where thoughtful criteria were applied to minimize, to the extent possible, the false positive rate. The analysis consisted of a pretreatment to eliminate uninformative data according to a calculated threshold; t = m + 2 × sd (where 't' is the calculated threshold, 'm' is the mean and 'sd' is the standard deviation of the negative control data, n = 424). Transcripts above the calculated threshold were considered as present in the bovine, mouse, or Xenopus laevis oocytes. A second independent analysis with the NIA Array Analysis tool was also conducted [53,54]. Briefly, raw data from the multi-species microarray hybridizations were uploaded and background threshold was determined according to the plot of error function (standard deviation, SD (= square root of the error variance), versus expression level (Log intensity)). Clones with a mean log signal intensity above the calculated background threshold (log 2.5) were considered as expressed in bovine, mouse, or Xenopus oocytes. Since both independent methods of analysis generated similar results, the list of genes expressed in the oocyte from the first method was used for the following steps.

#### Transcripts common in oocytes of all three species

Subsequently, clones for which all three species and all replicates were above the calculated threshold were selected to generate a list of transcripts common in oocytes of all three species (48 data points were generated for each clone; 4 spotted clones × 4 replicate hybridizations × 3 species).

#### Oocyte-specific transcripts common in all three species

To further characterize this subpopulation of transcripts common in oocytes of all three species, another classification was performed in order to identify the ones that are preferentially expressed in oocytes. This was done by comparing results from a previous study [24] where transcripts preferentially expressed in oocytes compared to somatic tissues were identified. In this previous study, a list of genes preferentially expressed in the oocyte was obtained through subtractive hybridization and microarray experiments in the bovine, mouse and Xenopus laevis. For the present study, the genes found to be conserved in all three species were compared against the list of genes preferentially expressed in the oocyte previously obtained. The combination of these two sets of results generated a list of oocyte-specific transcripts common in all three species.

#### Reproducibility

Data reproducibility was assessed by calculating the correlation coefficients between signal intensities across replicated experiments in a pair-wise manner with the NIA Array Analysis tool [53]. For each experiment, the signal intensities generated from a probe corresponding to one species was calculated by comparing the signal intensities obtained from a replicate experiment. The correlation coefficients were first calculated with the signal intensities of all clones on the microarray slides. Next, the correlation coefficients were calculated with the signal intensities of clones corresponding to one species at a time.

### Validation

To validate the presence of oocyte-expressed genes detected in all three species, RT-PCR analysis, using gene- and species-specific primers, were performed on three different selected candidate genes (Table 6). Briefly, equal amounts of total RNA isolated from bovine, mouse and Xenopus laevis oocytes was used to generate cDNA with an oligo (dT) primer and the Omniscript reverse transcriptase (Invitrogen) according to the manufacturer's instructions. PCR amplifications were performed as mentioned with gene- and species-specific primers in two species, not the species in which the candidate was first identified. Resulting amplicons were sequenced in order to check for specificity. Amplicons were then labeled as described above and used as probes to hybridize the multi-species microarray slide. Also, *GAPD *(Glyceraldehyde-3-phosphate dehydrogenase) amplifications were carried out as a positive control amplification to assure the quality of the cDNAs used for this experiment. *GAPD *PCR products were also labeled to use as a positive control of hybridization to assure the quality of the hybridization.

## Authors' contributions

MV designed, optimized and carried out the protocols, analyzed the results and wrote the manuscript. CR was involved in the conceptualization of this project and critically reviewed the manuscript for important intellectual content. SM was involved in the microarray data analysis and critically reviewed the manuscript. MFP provided support in the preparation of this manuscript and critically reviewed the manuscript for important intellectual content. MAS was involved in the conceptualization of this project, the preparation of the manuscript and provided mentorship. All authors read and approved the final manuscript.

## Figures and Tables

**Table 1:** Origin of clones conserved in all three species.

	Bovine oocyte library	Mouse oocyte library	Xenopus oocyte library	Total
Conserved in all three species	718	476	347	1541
Conserved in all three species and preferentially expressed in oocyte	120	96	52	268

**Table 2:** Clones preferentially expressed in oocyte and conserved across species.

Species^a^	Gene name (symbol)^bc^	GenBank Accession no^b^	% identity^b^	e value^b^
Bos taurus	Bt 675_bovine oocyte cDNA subtracted library	CX123770	100	0.0
Xenopus laevis	Xl AGENCOURT_10748348 Wellcome CRC pSK egg clone	CA974130	92	e-153
Bos taurus	Hs B-cell translocation gene 4 (*BTG4*)	NM_017589	89	0.0
Mus musculus	Mm Bone morphogenetic protein 15 (*Bmp15*)	BC055363	97	0.0
Mus musculus	Mm Expressed sequence C87414	BC052888	100	0.0
Bos taurus	Bt Growth differentiation factor 9 (*GDF9*)	AF307092	99	0.0
Bos taurus	Bt Hypothetical protein LOC616433	XM_881654	99	0.0
Mus musculus	Mm NACHT, leucine rich repeat and PYD containing 14 (*Nalp14*)	NM_001002894	98	0.0
Xenopus laevis	Xl NICHD_XGC_OO1 clone IMAGp998K1111210	BX843422	99	0.0
Mus musculus	Mm Nucleophosmin/nucleoplasmin 2 (*Npm2*)	NM_181345	98	0.0
Mus musculus	Mm Oogenesin 1 (*Oog1*)	AB050008	99	0.0
Bos taurus	Hs Polyadenylate binding protein-interacting protein 1 (*PAIP1*)	NM_183323	93	0.0
Xenopus laevis	Xl Protein arginine methyltransferase 1 (*PRMT1*)^d^	AB085173	99	0.0
Xenopus laevis	Xl Protein tyrosine phosphatase, receptor type Q (*PTPRQ*)	XM_370699	95	0.0
Mus musculus	Mm Similar to Nur77 downstream protein 1 (*Ndg1*)	XM_355193	99	0.0
Bos taurus	Bt Similar to zinc finger protein 678 (*ZF678*)	XM_601114	88	6e-68
Bos taurus	Bt Small fragment nuclease (*SMFN*)^d^	XM_591769	99	0.0
Mus musculus	Mm Spindlin (*Spin*)^d^	BC016517	100	0.0
Mus musculus	Mm Zona pellucida glycoprotein 2 (*Zp2*)	NM_011775	99	0.0

^a ^Species of the clone expressed in oocyte and conserved across species.

^b ^Based on the BLAST results of the clone sequences compared against the GenBank database.

^c ^Bt; Bos taurus, Hs; Homo sapiens, Mm; Mus musculus, Xl; Xenopus laevis.

^d ^Candidates validated by RT-PCR and gene-specific microarray.

**Table 3:** Correlation coefficients of the signal intensities between replicated experiments^a^

Species^b^	All clones^c^	Same-species^d^	Cross-species^e^		
			
			Bovine	Mouse	Xenopus
Bovine	0.957	0.973		0.965	0.923
Mouse	0.938	0.969	0.961		0.817
Xenopus	0.934	0.852	0.960	0.945	

^a^Results are presented as the average correlation coefficient.

^b^Corresponds to the species of the probe used for the hybridization.

^c^All spots on the microarray were considered irrespective of the species.

^d^Spots corresponding to the same species as the probe were considered.

^e^Spots corresponding to the other two species as the probe were considered.

**Table 4:** BLAST results for transcripts commonly expressed in oocytes of all three species^a^

	Bovine		Mouse		X. laevis	
Species^b^	Identity rate (%)	Length (nt)	Identity rate (%)	Length (nt)	Identity rate (%)	Length (nt)
Bovine			86_[73–96]_	259 _[32–721]_	80 _[73–90]_	304 _[41–1143]_
Mouse	86 _[81–94]_	188 _[48–458]_			81 _[74–90]_	149 _[42–323]_
X. laevis	80_[74–90]_	217 _[45–869]_	79 _[72–92]_	338 _[27–933]_		

^a ^The average identity rates is given in percentage (%) and the minimum and maximum in brackets. The average length is given in nucleotide (nt) and the minimum and maximum are in brackets.

^b ^Species of the transcripts compared against GenBank database.

**Table 5:** Transcripts that generated a significant positive hit when compared to GenBank database^a^

Species^b^	no hit^c^	1 hit^d^	2 hits^e^
Bovine	29%	71%	37%
Mouse	64%	36%	12%
X. laevis	41%	59%	48%

^a ^Significant positive hit when e value < 10 ^-5^.

^b ^Species of the transcripts.

^c ^The percentage of transcripts that generated no significant hits when compared to the GenBank database for the other two species.

^d ^The percentage of transcripts that generated a significant hit for at least one species.

^e ^The percentage of transcripts that generated a significant hit for both species.

**Table 6:** Sequence of gene-and species-specific primers.

Gene Name	Species	Primer Sequence	amplicon size
*Smfn*	Mouse	up 5'- TTT GTA CGA CAG CAG ACT CCT C -3'	245
		low 5'- TGA AGC TCT TTG ATG CTT TCA C -3'	
*SMFN*	Xenopus	up 5'- GAG CAC TGT GAA AGA ATT GTG C -3'	225
		low 5'- CTT CGA GTC AGA TGT TGG TCA C -3'	
*SPIN*	Bovine	up 5'- TGG CAT GGT CAT TCA TCA AG -3'	233
		low 5'- TTC TGC TGG CAG AGA TTG TG -3'	
*SPIN*	Xenopus	up 5'- CAG CCT TGT AGG GAA GCA AG -3'	184
		low 5'- TGT TTG GCA ACA AGT CAA GG -3'	
*PRMT1*	Bovine	up 5'- ACC CTC ACA TAC CGC AAC TC -3'	218
		low 5'- TTC CCC TTG ATG ATG GTC AC -3'	
*Prmt1*	Mouse	up 5'- ACC CTC ACA TAC CGC AAC TC -3'	208
		low 5'- TGA TGG TCA CCA CAT GGT CT -3'	
*GAPD*	Bovine	up 5'- CCA ACG TGT CTG TTG TGG ATC TGA -3'	226
		low 5'- GAG CTT GAC AAA GTG GTC GTT GAG -3'	
*Gapdh*	Mouse	up 5'- ATG TCG TGG AGT CTA CTG GTG TC -3'	486
		low 5'- CAR ACT TGG CAG GTT TCT CCA G -3'	
*GAPD*	Xenopus	up 5'- TGT AGT TGG CGT GAA CCA TGA G -3'	500
		low 5'- CAG CAT CAA AGA TGG AGG AGT G -3'	
